# 4326 Effects of Single-dose Preoperative Pregabalin on Postoperative Pain and Opioid Consumption in Cleft Orthognathic Surgery

**DOI:** 10.1017/cts.2020.125

**Published:** 2020-07-29

**Authors:** Abdullah Said, Ema Zubovic, Austin Y Ha, Gary B Skolnic, Jacob AuBuchon, Erika Waters, Kamlesh B Patel

**Affiliations:** 1 Washington University in St. Louis, Institute Of Clinical and Translational Sciences

## Abstract

OBJECTIVES/GOALS: The current opioid epidemic has placed post-operative pain management under scrutiny. Limiting post-operative pain can decrease overall opioid usage in the recovery period, especially after orthognathic surgery. Several studies have illustrated the efficacy of pregabalin in decreasing postoperative pain and opioid usage in adults undergoing orthognathic surgery. We aim to study the effects of a single dose of preoperative pregabalin on postoperative pain and total opioid consumption after orthognathic surgery in individuals with cleft lip and palate. METHODS/STUDY POPULATION: This was a retrospective cohort study of consecutive patients who received Le Fort I midface advancement between June 2012 and July 2019 by one of two surgeons at a single institution. We took advantage of our institution’s implementation, beginning in 2016, of a one-time dose of preoperative pregabalin for LeFort I midface advancement. All patients had diagnosed cleft lip and palate. The treatment group received a one-time preoperative dose of pregabalin. The control group did not receive pregabalin. Total morphine milligram equivalents (MME) consumption was calculated by adding intraoperative opioid administration and postoperative opioid consumption during admission. Postoperative pain control during admission consisted of oral oxycodone and intravenous (IV) hydromorphone or morphine. Duration of hospitalization and pain intensity assessed with the numeric pain rating scale (0-10) were also recorded. The mean postoperative pain assessment scores during admission was calculated for each patient. The median of these individual mean pain assessment scores for each group was subsequently computed. RESULTS/ANTICIPATED RESULTS: Twenty-three patients (14 males, 9 females) were included in this study; 12 patients received pregabalin (median dose: 150mg, range: 100-200mg). Mean age (years) at operation of the pregabalin (18.3 ± 1.9) and control groups (17.8 ± 1.9) were also equivalent (*p* = 0.571). Median hospital stay for both groups was 1.0 day. The pregabalin group had significantly lower consumption of total opioids during admission (total MME 70.95 MME; IQR: 24.65-150.17) compared to the control group (138.00 MME; IQR: 105.00-232.48) (MU = 31.00, *p* = 0.031). Although pain scores in the treatment group (3.21 ± 2.03) were lower than in the control group (3.71 ± 2.95), the difference was not statistically significant (*p* = 0.651, 95% Cl [−1.75, 2.75]). DISCUSSION/SIGNIFICANCE OF IMPACT: Based on the results, a one-time preoperative oral dose of pregabalin before orthognathic surgery in patients with cleft lip and palate reduced total opioid consumption during admission. However, there was no difference in length of stay or pain scores within the two groups. A single preemptive oral dose of pregabalin should be considered an effective adjunct to pain management protocols in patients undergoing orthognathic surgery.

